# Does faculty development influence the quality of in-training evaluation reports in pharmacy?

**DOI:** 10.1186/s12909-017-1054-5

**Published:** 2017-11-21

**Authors:** Kerry Wilbur

**Affiliations:** College of Pharmacy, Qatar University, PO Box 2713, Doha, Qatar

**Keywords:** Workplace-based assessment, Faculty development, Pharmacy

## Abstract

**Background:**

In-training evaluation reports (ITERs) of student workplace-based learning are completed by clinical supervisors across various health disciplines. However, outside of medicine, the quality of submitted workplace-based assessments is largely uninvestigated. This study assessed the quality of ITERs in pharmacy and whether clinical supervisors could be trained to complete higher quality reports.

**Methods:**

A random sample of ITERs submitted in a pharmacy program during 2013–2014 was evaluated. These ITERs served as a historical control (control group 1) for comparison with ITERs submitted in 2015–2016 by clinical supervisors who participated in an interactive faculty development workshop (intervention group) and those who did not (control group 2). Two trained independent raters scored the ITERs using a previously validated nine-item scale assessing report quality, the Completed Clinical Evaluation Report Rating (CCERR). The scoring scale for each item is anchored at 1 (“not at all”) and 5 (“exemplary”), with 3 categorized as “acceptable”.

**Results:**

Mean CCERR score for reports completed after the workshop (22.9 ± 3.39) did not significantly improve when compared to prospective control group 2 (22.7 ± 3.63, *p* = 0.84) and were worse than historical control group 1 (37.9 ± 8.21, *p* = 0.001). Mean item scores for individual CCERR items were below acceptable thresholds for 5 of the 9 domains in control group 1, including supervisor documented evidence of specific examples to clearly explain weaknesses and concrete recommendations for student improvement. Mean item scores for individual CCERR items were below acceptable thresholds for 6 and 7 of the 9 domains in control group 2 and the intervention group, respectively.

**Conclusions:**

This study is the first using CCERR to evaluate ITER quality outside of medicine. Findings demonstrate low baseline CCERR scores in a pharmacy program not demonstrably changed by a faculty development workshop, but strategies are identified to augment future rater training.

**Electronic supplementary material:**

The online version of this article (10.1186/s12909-017-1054-5) contains supplementary material, which is available to authorized users.

## Background

Health professional students participate in workplace-based training as a fundamental aspect of their education. Conducted in practice settings under the supervision of clinician mentors, students have opportunities to reinforce and shape development of existing knowledge and skills while engaged in direct patient care [1]. The in-training evaluation report (ITER) is a ubiquitous feature of this experiential training [2]. Also referred to as *field practicum*, *rotation*, or *clerkship* evaluations, these forms are documents of clinical supervisors’ judgements of student performance in patient care settings and serve as workplace-based assessment (WBA) instruments across health disciplines [3].

Despite programs’ reliance on an ITER as an account of trainees’ clerkship performance and collectively, as a reliable summative record of a student’s demonstrated skills, knowledge, and behaviours over time, rater variability pervades WBA and is typically considered undesirable [4]. Although most ITERs outline the student competency components to guide users, studies demonstrate that clinical supervisors do not uniformly interpret these descriptions or the accompanying defined rating scales, nor do they assign the same value to the performances expected to be evaluated [5, 6]. The standards against which they judge students are variable and include themselves, peers, and other trainees [7, 8]. Global student impressions may additionally shape specific domain scores indiscriminately; the mental workload required to process and score multiple dimensions further contributes to unconscious cognitive biases. [9, 10].

The ability of rater-training to effectively mitigate such influences on WBA is mixed [4, 11–13]. However, Dudek *el al* have demonstrated that ITER quality can be improved by both live workshop and asynchronous web-based faculty development which systematically emphasizes the written element clinical supervisors use to elaborate on physician trainee performance [14, 15]. A nine-item Completed Clinical Evaluation Report Rating (CCERR) scale was devised and validated by medical educators, including attending physicians who supervise students and residents [16]. ITER comments are rated according to the inclusion of both student strengths and weaknesses and the documentation of specific descriptions, recommendations for improvement, and the use of supportive language. Following initial in-person faculty development workshop, improvements in total CCERR scores reflected a moderate to large effect size and authors recommend the scale to others as a highly reliable objective measure of clinical supervisor evaluations [14].

Unlike medicine, the literature outlining the quality of documented student workplace-based performance in pharmacy is lacking. Direct patient care clerkships represent significant proportions of pharmacy curricula and merit the same audit that campus-based assessment strategies receive (e.g. course quizzes and exams, objective structured clinical examinations (OSCEs)) [17, 18]. Similarly, demonstrated evidence of direct patient care activities, assessment forms, and practice site feedback is expected of various accrediting bodies to ensure adequate intensity, breadth and structure of practice experiences [19, 20]. We hypothesize that the quality of narrative comments documented in pharmacy ITERs is deficient and as in medicine, may be improved through clinical faculty education. Herewithin, we report on the first experience using CCCER as a measure of ITER quality in pharmacy student experiential training and the effects of a professional development workshop.

## Methods

### Study design

A quasi-experiemental design using both retrospective and prospective controls was used to investigate the effectiveness of a faculty development workshop on the quality of narrative comments documented in pharmacy ITERs as measured by the CCERR instrument.

### Study Setting

The Doctor of Pharmacy (PharmD) program at the College of Pharmacy (CPH) at Qatar University (QU) is a Canadian-accredited post-baccalaureate degree supporting the training of graduate students to assume advanced pharmacy practice positions as integrated members of multidisciplinary teams delivering direct patient care. PharmD students complete 32 -weeks of experiential training (8 internships of 4 -weeks’ duration) for which ITERs are submitted by each internship’s clinical supervisor. The ITER used is a 25-item assessment instrument organized according to the seven domains of expected educational outcomes for graduating *medication therapy experts* (care provider, communicator, collaborator, manager, advocate, scholar, professional) and are akin to the CanMEDS framework for training the *medical expert* (physician) [19, 21, 22]. Assigned categorization for each described performance is *“exceeds expectations”; “meets expectations”;* or *“below expectations”* represented by 3, 2, and 1, respectively.

### Study Sample and Data Collection

The first cohort of ten graduate students enrolled in the QU CPH PharmD program in 2011. This small class size has fluctuated in number between 8 and 20 students over subsequent academic years. While students have the opportunity for overseas internships with various U.S. and Canadian university partners, the majority are offered locally by an expanding pool of nearly 50 clinical supervisors. To determine ITER quality in our program, we evaluated a random sample of those completed in the 2013–2014 academic year using the CCERR scoring tool (Additional file 1). On the annual rotation schedule, each student’s internship month was numbered in sequence (for example, student 1 (ordered alphabetically by surname) rotations numbered 1–8, student 2 rotations numbered 9–16, and so on). An online program generated a fixed number of random integers and the associated internship months selected for review. ITERs were blinded for clinical supervisor, student, and practice site by the author. Two pharmacist research assistants were trained and independently rated each ITER. Dudek et al. have repeatedly demonstrated that raters can reliably administer the CCERR without training beyond the instrument instructions [14, 16]. Nevertheless, we decided to review the CCERR instrument as a group to ensure shared understanding and met again following independent coding of the first two ITERs. Total CCERR scores for each ITER were calculated by adding the sum of values assigned to each item (all possible scores therefore ranged from 9 to 45). The aggregate CCERR score served as the comparative baseline group value (Control Group 1).

### Intervention

In the fall of the 2015–2016 academic year, a faculty development program was devised for all undergraduate and PharmD clinical supervisors similar in content to a workshop for medical trainee supervisors previously reported [14]. All local pharmacists who supervise and evaluate pharmacist trainee clinical internships were invited through direct emails and university announcements to participate in the workshop. The investigators led a structured discussion exploring roles of WBA tools and features of well-completed ITERs. Videos portraying (simulated) student patient encounters were shown and participants recorded their judgements in the relevant ITER sections. These resources were developed and shared with investigators by The Australasian College for Emergency Medicine [23].These narrative comments were then peer-reviewed in small groups using the CCERR tool and overall feedback for improvement shared by facilitators. Following the workshop, we then evaluated a blinded, random sample of PharmD student ITERs completed during the academic year (November 2015 to June 2016 inclusive) by those clinical faculty who attended the workshop (Intervention Group) and those who did not (Control Group 2). Random selection of ITERs followed the same process conducted for Control Group 1.

### Statistical analysis

The overall and weighted mean scores for each of the nine CCERR item categories in the intervention group were independently compared to both the historic and concurrent control groups using Mann-Whitney tests for non-parametric continuous data with significant level (2-sided) set at alpha 0.05. The proportion of CCERR item categories scored below the threshold score for acceptability (less than 3) was also calculated. Inter-rater reliability was calculated mid-way and at the conclusion of ITER review for the 2013–2014 and the 2015–2016 data sample. At the conclusion of ITER review of the 2013–2014 and of the 2015–2016 sample, each research assistant was asked to re-rate the same two randomly selected ITERs. Acceptable inter- and intra-rater reliability was set at 0.75 as measured by the intraclass correlation coefficient [24]. Ethics approval for each year of study conduct was obtained from the QU Institutional Review Board.

## Results

**Fig. 1:**
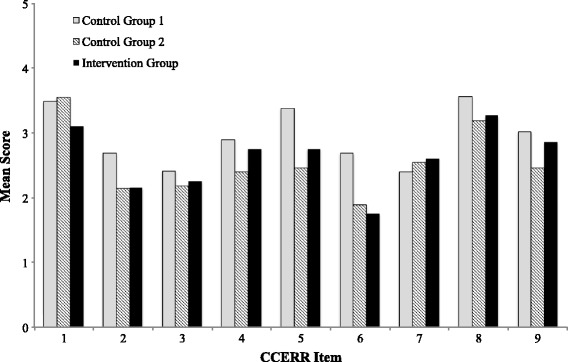
CCERR Item Scores for Pharmacy ITERs Before (Control Group 1) and Following (Control Group 2, Intervention Group) a Faculty Development Workshop

A random sample of 54 ITERs were reviewed as part of control group 1 (Fig. 1). The ability to clearly understand the student’s performance on the internship was rated acceptable for 37 (68.5%) of ITERs when total CCERR scores were considered. Mean scores for individual CCERR items were below acceptable thresholds for 5 of the 9 domains, including documented evidence of specific examples to clearly explain weaknesses (mean rating 2.48 ± 0.84) and concrete recommendations for student improvement (mean rating 2.26 ± 0.62) (Fig. 1). The aggregate mean CCERR score for this control group 1 was 37.9 ± 8.21.

Nine (19.6%) PharmD clinical supervisors attended the faculty development workshop and 77 student internships were subsequently completed during the remainder of the academic year (Table 1). All ITERs (10) submitted by participating clinical supervisors were reviewed (intervention group). A random sample of 14 ITERs completed by non-participating clinical supervisors (37, 80.4%) served as control group 2. When total CCERR scores were calculated, the overall ability to clearly understand the student’s internship performance was rated acceptable for 7 (50%) in the control group 2 and 7 (70%) in the intervention group (Fig. 1). However, mean scores for individual CCERR items were below acceptable thresholds for 6 and 7 of the 9 items, respectively, which represents more than that found in control group 1. There was no statistically significant difference in individual CCERR item scores when the intervention and control group 2 was compared, although 7 of the 9 category scores increased somewhat. When intervention group CCERR item scores were compared with control group 1, statistically significant decrease in item scores for clear documentation of examples of student strengths and weaknesses were found. The difference in aggregate mean CCERR score for the intervention group (22.9 ± 3.39) and control group 2 (22.7 ± 3.63) was not statistically significant (*p* = 0.84), but was found to be when each was compared to control group 1 (*p* = 0.0001). Interrater reliability between the two independent ITER assessors for the entire data set was 0.77. Overall assessor intrarater reliability was 0.87 and 0.91, respectively.

**Table 1:** Clinical Supervisor Demographics

	2013–2014 Academic Year	2015–2016 Academic Year	
	Control Group 1 *N* = 41	Intervention Group *N* = 9	Control Group 2 *N* = 37
Female	17 (41%)	6 (67%)	19 (51%)
ITERs completed^a^ (median, range)	2 (1–5)	1 (1–2)	2 (1–5)
New Preceptor	15 (37%)	5 (56%)	8 (22%)
Cumulative Supervisory Experience			
Students (median, range)	3 (1–7)	2 (0–7)	2 (1–5)
Years (median, range)	2 (0–3)	2 (0–2)	2 (0–4)

^a^In-training evaluation reports completed in the given academic year only

## Discussion

Ours is the first known study to use the CCERR scale as a measure of ITER quality in a health professional curriculum other than medicine. Clinical supervisor documentation of pharmacy student performance in workplace-based settings was considered below acceptable thresholds in most of CCERR item domains in both control groups. However, unlike improvements observed in medical student or residency training evaluations, negligible changes in ITER quality were demonstrated following a professional development workshop for pharmacist clinical supervisors.

As an instrument to determine ITER quality, the CCERR is unique, as to date no other tool for comparable use has been developed. While our findings could suggest that its utility outside medicine is poor given how health professions have distinct scopes of practice and student competencies to assess, the CCERR scale would appear suitably generic for application across disciplines. The majority of high-quality ITER features - identified by medical participants through focus group discussion, distilled into the nine CCERR items by Delphi consensus methodologies and subsequently validated - resonate with fundamental feedback principles for workplace-based learning, such as basing feedback on direct observation (e.g. specific examples of performance) and concluding with an action plan [25]. While we would not dispute the importance of confirming and facilitating student acceptance of feedback, it would not be a typical pharmacy program expectation for clinical supervisors to document the student’s response onto the ITER form. Instead, it may be reflected in clinical supervisors’ stated impression of the trainee’s overall attitude or professionalism [26]. Consequently, the related CCERR item score, documented trainee response to feedback and/or remediation was rated very low in our study.

Despite reinforcement through faculty development initiatives, shortcomings in the amount and nature of ITER narrative persist, especially as it pertains to constructive comments [27, 28]. In cases of poor performance especially, physicians have professed aversion to documenting even slightly negative feedback out of concern for a disproportionate effect on the trainee’s future career opportunities [29]. In our study, the CCERR item corresponding to the provision of specific descriptions for identified student weakness was rated lowest among control and intervention ITERs. Clinical supervisors in our setting may also share perspectives of the Canadians physicians previously described, but cultural factors may be additionally implicated. Members of high context societies (as Arab countries like Qatar are often characterized) exhibit preferences for nuanced non-verbal communication and may therefore be reluctant to record feedback, especially comments that may be interpreted as overly critical [30]. Such preferences for verbal communication may have contributed to the low scores on most other CCERR items. If the ITER is additionally considered a surrogate marker for feedback exchange in the workplace-based setting, it may have been worthwhile to triangulate our data collection with trainee interviews following the faculty development workshop to determine if they recall receiving verbal feedback from their clinical supervisors to help further discriminate the numeric scores of rated skills and performances recorded on their ITER. Although English is the predominant language of care delivery in Qatar, our clinical supervisors, many of who are of Arab-origin, complete ITERs in their second language (English). We do not know if a notion or idea for a commentary about the trainee occurs to an Arab clinical supervisor in their native language, but then not translated to English for ITER documentation, further confounding culturally-oriented barriers to documented narrative evaluation.

As an objective measure of quality, the CCERR scale still relies on subjective assessment (not unlike a patient’s self-reported pain scale, for example). Use of multiple independent raters for CCERR administration may mitigate this phenomenon, but inherent cognitive biases invariably persist, especially as it pertains to language use and interpretation [31, 32]. In our study, CCERR item scoring the supportive nature of ITER comments was among those items rated lowest, but admittedly, we may not have exhausted potential examples among our research assistants. Is advocated clinical supervisors use of specific neutral language focusing on performance in ITER documentation incompatible with support or encouragement and therefore this CCERR item? [25]. Prior work suggests written comments in ITERs are actually often vague and subject to ‘decoding’ by the reader [33]. Indeed, linguistic analysis of the non-literal language found in Canadian medical ITERs detected the presence of politeness strategies, such as ‘hedging’, whereby a word is used to lessen the impact of the an utterance (or in the case of an ITER, the impact of the written message) [34]. These readers also interpreted clinical supervisors’ use of verbs indicating change (e.g. improving, developing) as having negative implications and considered a “good” resident below average. It is unclear then how CCERR scoring among distinct professions and contexts may be influenced by rater understanding of language used.

Our orphan assessment of ITER quality in pharmacy education is concerning. The medical literature is replete with study of the validity and reliability of the ITER numeric scoring component and increasingly, scrutiny of narrative comment but thus far our work is without apparent precedent in any other health profession. [14–16, 29, 35]. Experiential training programs use submitted ITERs to capture student demonstrated day-to-day abilities as important data points to determine student achievement of expected competencies and ultimately fitness to graduate and eligibility for professional licensure. Comments may serve as more useful clues than scores in identifying students in difficulty. [36]. Quality assurance findings in health professional training experiences may also serve as a surrogate marker of communication between the clinical supervisor and student as it pertains to constructive feedback and supportive reinforcement. The ITER serves as a tool for formative assessment documented at the midpoint and conclusion of the student’s clerkship with each clinical supervisor. Perceived student progress in demonstrating achievement of the seven main educational outcomes of a *medication expert* are formally discussed and recorded. Irrespective of undocumented verbal feedback exchanges between trainer and trainee throughout this learning experience, our program expects suitable support of the summative judgement of student performance and ultimately clerkship outcome (to pass or to fail). We have found through the low CCERR scores that this narrative element of ITER completion among our clinical supervisors can be improved. We encourage adoption of systematic ITER quality assurance processes for pharmacy programs and other health disciplines.

Although our absolute aggregate mean CCERR scores are in fact higher than those found following ITER faculty development among physicians, the small number of completed ITERs considered for evaluation following the faculty development workshop, especially in the intervention group, is a limitation to our findings [14]. Inherent high quality ITERs in our first control group may have minimized the ability to detect a difference following the workshop. However, even within this cohort of randomly selected ITERs, the majority of CCERR items were still scored less than the acceptable threshold. Additionally, clinical supervisors demonstrating enhanced written feedback on ITERs within an undistracted workshop setting may not be able to replicate the protected time in busy clinical settings. In analysis of control group 1, we detected the ITER layout may itself restrict the capacity for documenting narrative feedback [37]. However, our greatest failing in demonstrating improvements in ITER quality following the faculty development workshop is quite likely our lack of follow-up with feedback. In subsequent study, the CCERR developers created a distance-based faculty development program whereby participants received biannual feedback on the quality of their submitted ITERs over 2 years [15]. Similar reinforcement of written feedback principles over time may also result in improved ITER quality in our pharmacy clinical supervisors [38].

## Conclusion

The quality of narrative comments documented in pharmacy ITERs as measured by the CCERR instrument was low and did not appreciably change following a faculty development workshop. Despite our first experience, we believe the CCERR instrument can be a useful vehicle to support improved WBA documentation in pharmacy student workplace-based training. The challenges to securing professional development attendance is widely acknowledged and our program is also exploring distance-based faculty development models that would incorporate routine personalized ITER feedback to clinical supervisors [15]. Greater understanding of the barriers clinical supervisors face in documenting assessment of health professional trainees is necessary. In future study, we will also further explore the applicability of CCERR scoring of ITERs in distinct cultural contexts.

## Additional file

Additional file 1: Completed clinical evaluation report rating (CCERR) – Final scale. Instrument used to rate In-Training Evaluation Reports (ITERs) in our study. (DOC 46 kb)

## Abbreviations

CCERR: Completed Clinical Evaluation Report Rating
CPH: College of Pharmacy
ITER: In-training evaluation reports
QU: Qatar University
WBA: Workplace-based assessments
