# Supplementary material for: Does faculty development influence the quality of in-training evaluation reports in pharmacy?
Source: BMC Med Educ. 2017 Nov 21;17:222. doi: 10.1186/s12909-017-1054-5 (PMC5697106; doi:10.1186/s12909-017-1054-5)
Supplement: Additional file 1: — Completed clinical evaluation report rating (CCERR) – Final scale. Instrument used to rate In-Training Evaluation Reports (ITERs) in our study. (DOC 46 kb) [file 12909_2017_1054_MOESM1_ESM.doc]

**Appendix 1 - Completed clinical evaluation report rating (CCERR) – Final scale**

The purpose of this scale is to evaluate the quality of how a clinical evaluation form, such as an In-Training Evaluation Report (ITER) has been ***filled out***. With this in mind, please use this scale with regard to ***how the form has been completed*** rather than the design of the form.

“Ratings” refers to the checklists or global ratings used on the evaluation form.

If there is no space for comments on the form, this scale cannot be used. However, if there is a comments section but no comments have been included (i.e. the comments section is blank), please indicate a score of “1” for the questions referring to the comments.

Please rate the following by checking the appropriate box.

|  |  | **1**  **Not at all** | **2** | **3**  **Acceptable** | **4** | **5**  **Exemplary** |
| --- | --- | --- | --- | --- | --- | --- |
| 1. | **Checklist/numeric ratings** show sufficient variability to allow identification of **relative** strengths and weaknesses of the trainee. |  |  |  |  |  |
| 2. | Comments are **balanced** providing both strengths **and** areas for improvement. |  |  |  |  |  |
| 3. | The trainee’s response to feedback and/or remediation during the rotation is described in the comments. |  |  |  |  |  |
| 4. | Comments justify the ratings provided. |  |  |  |  |  |
| 5. | Clearly explained examples of strengths using specific descriptions (not generalizations) are provided in the comments. |  |  |  |  |  |
| 6. | Clearly explained examples of weaknesses using specific descriptions (not generalizations) are provided in the comments. |  |  |  |  |  |
| 7. | Concrete recommendations for the trainee to attain a higher level of performance are provided. |  |  |  |  |  |
| 8. | Comments are provided in a supportive manner. |  |  |  |  |  |
| 9. | Overall, this ITER provides enough detail for an independent reviewer to clearly understand the trainee’s performance on the rotation. |  |  |  |  |  |

Copyright permission for use obtained from Medical Education
